# Generating Practice-Based Evidence in the Use of Guideline-Recommended Combination Therapy for Secondary Prevention of Acute Myocardial Infarction

**DOI:** 10.3390/pharmacy10060147

**Published:** 2022-11-03

**Authors:** Mary C. Schroeder, Cole G. Chapman, Elizabeth A. Chrischilles, June Wilwert, Kathleen M. Schneider, Jennifer G. Robinson, John M. Brooks

**Affiliations:** 1 Division of Health Services Research, College of Pharmacy, University of Iowa, Iowa City, IA 52242, USA; 2 Department of Epidemiology, College of Public Health, University of Iowa, Iowa City, IA 52242, USA; 3 Schneider Research Associates, Des Moines, IA 50312, USA; 4 Center for Effectiveness Research in Orthopaedics, Arnold School of Public Health, University of South Carolina, Columbia, SC 29208, USA

**Keywords:** myocardial infarction, combination drug therapy, Medicare claims analyses, selection bias, unmeasured confounders, comparative effectiveness research

## Abstract

**Background**: Clinical guidelines recommend beta-blockers, angiotensin-converting enzyme inhibitors/angiotensin-receptor blockers, and statins for the secondary prevention of acute myocardial infarction (AMI). It is not clear whether variation in real-world practice reflects poor quality-of-care or a balance of outcome tradeoffs across patients. **Methods**: The study cohort included Medicare fee-for-service beneficiaries hospitalized 2007–2008 for AMI. Treatment within 30-days post-discharge was grouped into one of eight possible combinations for the three drug classes. Outcomes included one-year overall survival, one-year cardiovascular-event-free survival, and 90-day adverse events. Treatment effects were estimated using an Instrumental Variables (IV) approach with instruments based on measures of local-area practice style. Pre-specified data elements were abstracted from hospital medical records for a stratified, random sample to create “unmeasured confounders” (per claims data) and assess model assumptions. **Results**: Each drug combination was observed in the final sample (N = 124,695), with 35.7% having all three, and 13.5% having none. Higher rates of guideline-recommended treatment were associated with both better survival and more adverse events. Unmeasured confounders were not associated with instrumental variable values. **Conclusions**: The results from this study suggest that providers consider both treatment benefits and harms in patients with AMIs. The investigation of estimator assumptions support the validity of the estimates.

## 1. Introduction

There have been considerable changes in the management of acute myocardial infarction (AMI) over the last three decades, along with decreasing rates of hospitalization and mortality [1,2,3]. Even so, an estimated 605,000 new and 200,000 recurrent attacks will occur in the United States this year, approximately one every 39 s [4]. For patients who survive the AMI, the American College of Cardiology and American Heart Association guidelines recommend beta-blockers (BB), angiotensin-converting enzyme inhibitors/angiotensin-receptor blockers (AA), and statins (ST) following hospital discharge for secondary prevention [5,6]. Despite this, most patients do not receive guideline-recommended treatment (BB+AA+ST) [7,8,9,10,11,12,13,14,15,16,17,18,19,20,21,22,23]. Indeed, the use of *all* possible combinations of these drugs have been observed in real-world practice [13].

However, this variation may not be completely unfounded. Almost four decades have passed since the publication of the first BB trials, which themselves preceded the implementation of acute coronary revascularization and widespread ST use [13,24,25,26]. In addition, none of the randomized controlled trial (RCT) designs included head-to-head comparisons against the use of all three drugs, let alone across all eight possible combinations of these drugs [5,16,26,27,28,29,30,31]. The external validity and generalizability of the trials have been challenged over the last 20 years, as older patients, women, and those with comorbidity were routinely excluded [32,33,34,35,36,37,38]. Deviations from guideline-recommended care could be something other than poor quality-of-care. It may be the result of “practice-based evidence” where providers considered the clinical heterogeneity across patients in addition to guidelines when making treatment recommendations, and, over time, became aware of outcome tradeoffs that vary with patient circumstances when prescribing secondary prevention treatments for patients with AMIs [39,40,41,42,43,44,45,46].

Unfortunately, new RCTs to address these known knowledge gaps are unlikely. Not only are the medications readily available as lower-cost generics, but randomization to any treatment combination other than BB+AA+ST would be contrary to the well-established guidelines, and bring forth ethical questions of equipoise. Considering this reality, observational studies remain as an alternative approach to assessing the effectiveness of alternative combination therapies. Previous research using retrospective data report higher overall survival rates for AMI patients using guideline-recommended treatments [13,16,47,48]. However, these studies did not evaluate possible detriments, such as adverse events, which may be associated with guideline-recommended treatments. In addition, the risk adjustment methods used in these studies yield unbiased estimates under the assumption that unmeasured factors affecting patient outcomes (e.g., disease severity and functional status) are unrelated to treatment choice [49,50,51,52,53,54,55,56,57]. The validity of these assumptions were not examined in these studies.

The objective of this study to generate practice-based evidence associated with higher rates of guideline-recommended treatment for AMI patients in real-world practice. One-year overall survival, one-year cardiovascular-event-free survival, and 90-day adverse events were assessed for a sample of Medicare fee-for-service beneficiaries hospitalized in 2007–2008 with AMI. An instrumental variables estimator was used that followed the approaches in previously published studies [58,59]. The instruments specified in this study reflect “treatment signatures”, which have been described as the idiosyncratic clinical rules-of-thumb developed by providers within a local area [60,61,62,63,64,65]. This choice of instrument ensures that parameter estimates are directly interpretable as the change in outcome rates associated with higher treatment rates. In addition, an evaluation of IV estimator assumptions was incorporated into the study design from its inception [66]. Prior to any data extraction, an extensive list of known and theorized confounders was established with input from the scientific literature, clinical experts, and economic theory [50,52,53,54]. Confounders able to be measured using Medicare data were directly controlled for in IV estimation. The remaining “unmeasured confounders” were measured using from abstracted medical-records data for a stratified, random sample of the final study cohort, and used to assess IV estimator assumptions.

## 2. Methods

### 2.1. Data, Cohort Selection, and Model Covariates

Medicare claims data obtained from the Chronic Condition Data Warehouse were used to identify all Medicare fee-for-service beneficiaries within the continental United States (lower 48 states) hospitalized with an AMI in 2007 or 2008. Data for these beneficiaries included enrollment information, final adjudicated Part A and Part B medical claims, and Part D prescription drug events. The date of acute hospital admission with a principal diagnosis of AMI served as the index admission date for the patient. The index stay was defined to include all contiguous days of care at any/all inpatient institution(s), beginning with the initial acute hospitalization. The end date for the index stay was the date the patient was finally discharged home, and was calculated by combining all Medicare institutional claims (acute, long-term care hospital, inpatient rehabilitation facility, critical-access hospital, and skilled nursing facility) with overlapping admission and discharge dates following the initial admission [67]. Patients were excluded from the study cohort if they had any diagnosis of AMI in Part A or B claims in the 12 months prior to the index admission date, were not at least 66 years old at the time of index admission, were not continuously enrolled in Medicare fee-for-service Part A and B from 12 months prior to the index admission date through 12 months after the index discharge date or until death (whichever occurred first), were not continuously enrolled in a Part D Prescription Drug Plan from the 12 months prior to the index date through 6 months after the index discharge date or death (whichever occurred first), or did not reside in the contiguous lower 48 states. To ensure accurate observation of medication use, patients enrolled in a hospice, admitted for inpatient care or to a skilled nursing facility [67], or who died within 30 days of being discharged home from the index stay were also excluded.

#### 2.1.1. Treatment Measures

This study focused on 3 medication classes recommended by clinical guidelines for secondary prevention following an AMI: beta-blockers (BB), angiotensin-converting enzyme inhibitors/angiotensin-receptor blockers (AA), and statins (ST) [5,6]. From these, patients were grouped into 1 of 8 possible treatment combinations: none, BB alone, AA alone, ST alone, BB+AA, BB+ST, AA+ST, or BB+AA+ST. Drugs were identified by linking the National Drug Codes on Part D event claims to medication classes using Multum Lexicon software. Treatment variables were specified for each patient, and set to 1 if the patient had the drug available for use in the 30 days following discharge from the index stay, 0 otherwise. A drug was designated as available to a patient if a Part D event occurred for that drug within 30 days post-discharge or if the patient had a sufficient supply “on the shelf” at home at time of discharge to cover the 30 days post-discharge. “On the shelf” supply was calculated based on “days supplied” information from Part D events filled before the discharge date.

#### 2.1.2. Outcome Measures

The primary outcomes of interest were one-year overall survival, one-year cardiovascular-event-free (CVE-free) survival, and adverse events occurring within 90 days of discharge. Cardiovascular events included another AMI, unstable angina, or stroke. Adverse events were defined as the presence of an inpatient claim with diagnoses for hypotension, bradycardia, heart block, angioedema, hyperkalemia, serious myopathy, an acute renal event, or an acute hepatic event. The specific medical coding terminology used to identify these outcomes can be found in previously published work [58,59].

#### 2.1.3. Model Covariates

Covariates and confounders measurable from Medicare claims and administrative data were identified *a priori* by the study team for the IV analyses. All models controlled for demographic variables (e.g., age, race); primary AMI diagnosis for the initial acute hospitalization (e.g., anterior wall); baseline medical history and comorbidity pre-AMI and during hospitalization (e.g., unstable angina, cardiac arrest, atrial fibrillation, ischemic heart disease, complicated hypertension); procedures pre-AMI and during hospitalization (e.g., coronary artery bypass grafting, coronary stent, cardiac catheterization); medication use 180-days pre-AMI (e.g., warfarin, insulin, calcium-channel blocker, diuretic); potential contraindications to study drugs pre-AMI and during hospitalization (e.g., hypotension, hyperkalemia, myopathy, chronic kidney disease); healthcare utilization during hospitalization (e.g., days in intensive care unit, days in cardiac/coronary care unit, emergency room use); insurance variables (e.g., low-income subsidy, dual-eligible) and financial burden at time of discharge (e.g., Part D benefit phase, cumulative beneficiary responsibility amount); and urbanicity and socioeconomic (SES) variables (e.g., residence in a metropolitan area, residence in an area with above-median percent living in poverty). The complete list of model covariates is further detailed in File S1.

### 2.2. Modeling Framework and Statistical Analyses

#### 2.2.1. Assumptions and Specifications of Estimation Framework

IV estimators generate unbiased estimates of treatment effects under the assumption that the specified instrumental variables are unrelated to unmeasured confounders [49,55,68,69,70]. IV parameter estimates are generalizable to the subset of patients whose treatment choices were sensitive to the values of the instrument, i.e., the “marginal patients” [71,72,73,74]. This interpretative framework is particularly appropriate in assessing the real-world comparative effects of higher rates of guideline-recommended treatments for secondary prevention. Two-stage least squares (2SLS) IV estimators were used in this study. The 2SLS estimators produce a consistent estimate of the absolute Local Average Treatment Effect regardless of the distribution of the underlying error term [75]. In addition, 2SLS parameter estimates are normally distributed with large sample sizes, per the central limit theorem [76]. The 2SLS estimators have been shown to provide consistent estimates of the treatment effect parameters under minimal assumptions, are preferable over nonlinear two-stage IV estimators with a binary dependent variable [77], and produce coefficients interpretable in terms of absolute change.

The identification strategy in this study is based on prior research developing instrumental variables from variation in treatment rates across geographic areas [78,79]. This strategy is particularly appropriate to assess the outcomes associated with higher-rates of guideline-recommended treatment as the instruments, based on Area Treatment Ratios (defined below), which are, themselves, measures of risk-adjusted, local-area rates of treatment choice. Variation in these instruments has been theorized to reflect differences in provider practice styles or “treatment signatures” across local areas. [58,74,79,80,81,82,83,84,85,86,87,88,89,90,91,92,93,94,95,96].

As with any modeling framework, there are assumptions that must be made and deemed reasonable. For the identification strategy of this study to be valid as a quasi-natural experiment in treatment choice, the variation in risk-adjusted average-treatment-rates across areas must only reflect differences in provider practice-styles that are not otherwise related to A) outcomes or B) unmeasured patient characteristics that affect outcomes. *A priori*, these assumptions seemed valid to the study team, as there is no reason to believe that patients select their residential ZIP code based on an area’s tendency to use BB+AA+ST for the secondary prevention of AMI. In this study, we provide *ex post* assessment of these assumptions.

To create the instruments in this study, an Area Treatment Ratio (ATR) for each of the drug combinations was estimated for the local area surrounding each residential ZIP code of every patient in the study cohort, and was defined as the ratio of the number of patients in their local area who received a particular drug combination over the sum of the predicted probabilities of those same patients receiving that same drug combination. The predicted probabilities were estimated across the entire (national) study cohort using a logistic regression model adjusting for all measured covariates. “Local area” was defined to include all patients residing in that ZIP code, along with the patients in each of the sequentially nearest ZIP codes (by driving distance) necessary to satisfy the requirement that the local area include a minimum of 150 patients [78,95]. By construction, ATR values are continuous, strictly positive, and distributed around the value 1 [87]. An ATR value >1 indicates greater use of a particular drug combination in that local area than would be predicted nationally. ATR values < 1 indicate the opposite: less use in an area than predicted nationally. A step function was used to create quintiles of the ATR values, and this set of dichotomous variables were the instruments specified in the IV models. By design, then, patients residing in the local areas assigned to the first (bottom) quintile of the ATRs for a particular drug combination were those least likely to have that drug combination, compared to similar patients residing in local areas grouped in the higher quintiles. Additional details are provided in File S2.

#### 2.2.2. Statistical Analyses

Patients who received guideline-recommended therapy (BB+AA+ST) were set as the reference group for the analyses. First-stage treatment choice regressions were estimated individually for the remaining 7 drug combinations. These first-stage treatment-choice models were specified as a function of the instruments and all measured covariates. F-statistics were used to assess the correlation between the instruments and treatment choice in this first-stage model. For the second-stage model, study outcomes were regressed on the estimated treatment probabilities for each of the 7 drug combinations, along with the same set of measured covariates included in the first-stage model.

All models were estimated with robust standard errors. The analytic dataset was created using SAS software (version 9.3; SAS Institute Inc.; Cary, NC, USA), and statistical analyses were performed with Stata (version 17; StataCorp LLC.; College Station, TX, USA). An Institutional Review Board approved this study.

### 2.3. Evaluating IV Assumptions Regarding Unmeasured Confounders

This study was designed, from its inception, to include a set of confounders unmeasurable with respect to the Medicare data, but measurable using medical records data, for the express purpose of evaluating IV model assumptions. To complete this task, the study team created a structured data-abstraction tool to capture additional, “unmeasured” clinical-assessment and treatment information. Concepts from validated instruments used in the Cooperative Cardiovascular Project [97] and Women’s Health Initiative [98] were also included. Variables were modified and customized in consultation with study team cardiologists, internists, and nurses. The study team then developed a Microsoft Access application with a user-friendly, front-end interface to ensure accurate and consistent abstraction of the necessary data elements. This tool was field tested, and inter-rater reliability scores were calculated for each data element across abstractors prior to implementation. With permission and approval from the Centers for Medicare & Medicaid Services (CMS), a contracted, third-party “honest broker” obtained copies of medical records from the index stay for a stratified, random sample of the final study cohort, and extracted the pre-specified data elements using the electronic abstraction tool. Additional details of the design, development, testing, and protocol in abstracting and creating these data are previously published [66], and a summary is provided in File S3.

Broadly speaking, the unmeasured confounders commonly cited in comparative effectiveness studies of cardiovascular drugs [50,52,53,54] reflect both the potential benefit of treatment (i.e., risk of recurrent AMI) and potential harm from treatment (i.e., risk of treatment-related adverse events). Specific to this study, the abstracted medical records data were used to create proxies of the disease burden and severity of AMI (Table S1), as well as identify potential contraindication to the study drugs. Functional status, another commonly cited unmeasured confounder, was proxied by abstracting data elements to calculate difficulty with activities of daily living (ADLs) [99,100] and the Adult Comorbidity Evaluation 27 (ACE-27) score [101,102,103]. Finally, height and weight information were abstracted to calculate BMI and identify underweight (BMI < 18.5) and overweight (BMI > 25) patients. An additional variable was created to proxy hospital quality: cardiac catheterization performed within 24 h of admission. Although the procedure itself can be identified from claims data, the timing of the procedure is not available.

An Ordinary Least Squares model was fitted to test for trends in these unmeasured confounders across treatment choice and instruments. Statistically significant test statistics across drug combinations would indicate selection bias. Statistically significant test statistics across the ATR quintiles would indicate a possible violation of IV model assumptions and bias in the associated parameter estimates.

## 3. Results

### 3.1. Study Population: Characteristics, Treatment, and Outcomes

The final cohort included 124,695 hospitalizations for individual patients with an AMI in 2007 or 2008 (Table 1). The median age at time of admission was 78 years old, with 20.6% of the study cohort 86 or older. Common comorbid conditions included uncomplicated hypertension (81.5%), hyperlipidemia (66.9%), and ischemic heart disease (56.5%). Almost half of the sample had two or more of the conditions listed in the Charlson Comorbidity Index. Within 30 days post-discharge, 73.9% had a BB, 56.8% had an AA, and 60.7% had a ST. Guideline-recommended treatment (BB+AA+ST) was observed in 35.7% of this population, and 13.5% had none of the study drugs. More-intense therapy (i.e., a greater number of distinct drugs in combination) was observed in younger patients, healthier patients (as measured by the Charlson Comorbidity Index), and those with more severe AMI (all *p*-value < 0.001). Unadjusted one-year overall and CVE-free survival were 84.3% and 75.4%, respectively, for the study cohort, and 5.4% experienced an adverse event within 90 days of discharge. Survival tended to increase with the number of drugs, whereas, perhaps paradoxically, adverse events trended lower with the number of drugs.

### 3.2. Instrumental Variables (IV) Analyses

**Figure 1:**
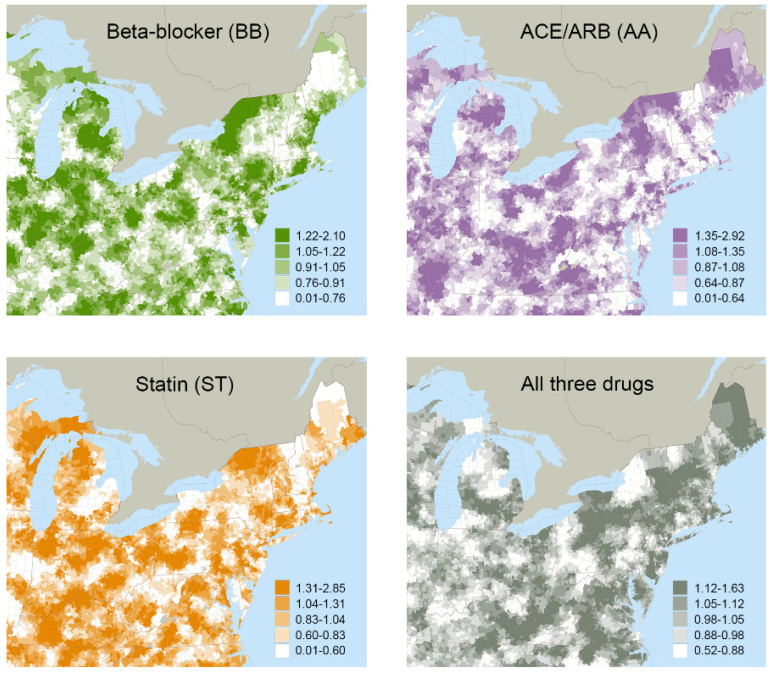
Quintiles of Area Treatment Ratio (ATR) Values Mapped for Four of the Possible Drug Combinations.

The mean treatment rates for local areas grouped by ATR quintiles are reported in Table 2. Variations in treatment rates were observed across quintiles, as well as geographically, for the various drug combinations (Figure 1). For example, 35.7% of the study cohort had guideline-recommended treatment (BB+AA+ST) overall, but this varied from a mean of 27.7% in the local areas grouped in the lowest quintile to 42.8% in the local areas grouped in the highest quintile. First-stage F-statistics ranged from 29.0 to 34.5 across all drug combinations, satisfying traditional thresholds (>10) to support that the instruments were not weak [104,105].

**Figure 2:**
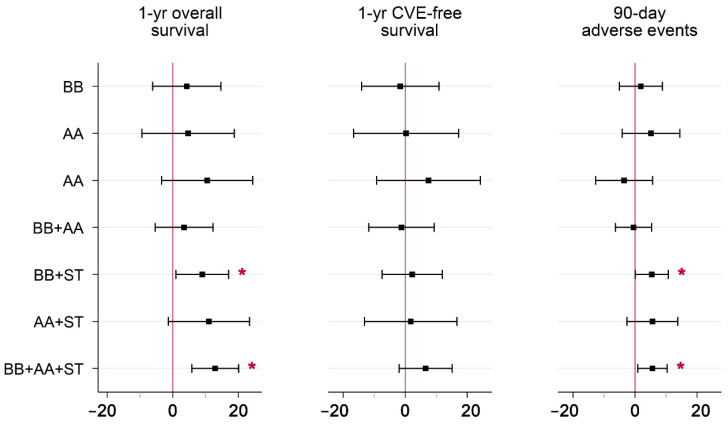
Parameter Estimates from IV Models of the Comparative Effectiveness of Various Post-discharge Drug Combinations on Positive and Negative Outcomes*. Abbreviations: IV = instrumental variables; AMI = acute myocardial infarction; CVE = cardiovascular event; BB = beta-blocker; AA = ACE/ARB; ST = statin. * Guideline-recommended combination therapy (BB+AA+ST) was set as the reference group. Parameter estimates reflect absolute effects, and are measured in percentage points. Whiskers include the 95% confidence intervals for each estimate. Mean (full sample) outcomes were 84.3% for overall survival, 75.4% for CVE-free survival, and 5.4% for adverse events. Covariates included in the models are listed in File S1.

IV parameter estimates for the seven non-guideline drug combinations relative to guideline-recommended combination therapy are reported in Table 3 and plotted in Figure 2 for each of the three outcomes. Several parameter estimates were significant at the 5% level. Of these, lower one-year survival rates were observed in local areas with higher rates of BB+AA, BB, and no drugs, relative to guideline-recommended combination therapy. Lower 90-day adverse event rates were observed in local areas with higher rates of BB+AA, ST, and no drugs, relative to guideline recommended combination therapy. Of the 21 parameter estimates, 19 were negative. Since both beneficial (overall survival and CVE-free survival) and detrimental (90-day adverse events) outcomes were analyzed, consistently negative estimates indicate a tradeoff in treatment, i.e., increased rates in the use of certain non-guideline-recommended drug combinations were associated with both poorer survival *and* a lower risk of adverse events, compared to BB+AA+ST.

### 3.3. Evaluating IV Assumptions Regarding Unmeasured Confounders

Of the 1404 patients in the stratified, random sample of the study cohort, 67.0% were overweight (BMI > 25), and 25.7% had difficulty in at least one activity of daily living (ADL), with 7.0% dependent on others for care in two or more domains (Table S2). Less than half (39.2%) underwent cardiac catheterization within 24 h of admission. Troponin levels exceeded 1.0 for most patients (65.0%) at some point of their initial hospitalization, and 12.5% were current smokers (Table S3). During the stay, 37.4% had low density lipoproteins (LDL) >100 (out-of-range, high), and 51.4% had high density lipoproteins (HDL) <40 (out-of-range, low). Almost half (46.3%) had a potential contraindication to the study drugs listed in the medical record at the time of hospital discharge; most common were a diagnosis of acute renal failure while hospitalized for AMI (16.5%), findings of pulmonary hypertension on echocardiograms performed during the stay (13.7%), and a history of chronic kidney disease (18.7%) or chronic obstructive pulmonary disease with dyspnea (17.1%).

Statistically significant trends were observed in unmeasured confounders across drug combination groups (Table 4). More-intense therapy (i.e., a greater number of distinct drugs in combination) was associated with more-severe AMI, a greater burden of disease, being overweight, or undergoing cardiac catherization within 24 h of admission (all *p* < 0.001). More-intense therapy was also associated with healthier patients, as measured here by lower ACE-27 scores and less difficulty with ADLs, as well as a lower likelihood of having a potential contraindication to the study drugs (all *p* < 0.001). In contrast, there was no association between these same unmeasured confounders and the instruments. Table 5 reports the summary statistics of these variables across ATR quintiles of guideline-recommended treatment (BB+AA+ST). Similar patterns (i.e., no significant trends) were observed across ATR quintiles for the other drug combinations (results not shown).

## 4. Discussion

Guidelines recommend that patients receive beta-blockers, angiotensin-converting enzyme inhibitors/angiotensin-receptor blockers, and statins (BB+AA+ST) for the secondary prevention of AMI [5,6]. In this study of fee-for-service Medicare beneficiaries hospitalized for AMI in 2007–2008, only 35.7% had all three drugs within 30-days of discharge, and the use of all eight possible drug combinations was observed. Our findings are consistent with others showing large deviations from clinical guidelines [7,8,9,10,11,12,13,14,15,16,17,18,19,20,21,22,23]. In this study, an IV approach was applied to observational Medicare claims data in this study to assess whether higher rates of guideline-recommended therapy would benefit patients without additional risks. Results from the IV models indicate a tradeoff between the beneficial and detrimental outcomes associated with a greater use of guideline-recommended treatment, where higher rates of BB+AA+ST were associated with better overall-survival and more adverse-events. Additionally, an assessment of IV model assumptions was planned from the project’s inception, and hospital medical records were abstracted for a stratified, random sample to collect information unavailable in the Medicare claims data. The unmeasured confounders evaluated in this study were randomly distributed across instrument values, supporting the validity of our IV estimation approach.

Our findings suggest that the observed deviation from guideline-recommended treatment in real-world practice may not necessarily indicate poor quality-of-care or physician ignorance. Higher rates of guideline-recommended treatment were associated with both beneficial *and* detrimental effects in this population-based sample. Given these tradeoffs, it is possible that existing treatment rates reflect practice-based evidence, where physicians are not only asking “does X cause Y?”, but also “how will adding X alter the complex personalized system of the actual patient before me?” [39]. Such practice requires a complex calculus that simultaneously considers the clinical heterogeneity of patient population intervention combinations, and outcomes to yield treatment recommendations for individual patients [40,41,42,43,44,45,46].

The results from other work also suggest patient-centered treatment decisions and the use of practice-based evidence. In two previous papers, 62% of patients surviving an AMI had a statin available within 30 days of discharge, far below what was recommended by clinical guidelines [91,106]. In these studies, lower statin-use rates were observed for patients with complex comorbidities and a history of adverse events known to be correlated with statin use in population-based studies [107,108]. Substantial geographic variation in treatment rates was also observed [91], and physician-specific statin prescribing-rates were normally distributed around the population mean, suggesting that physicians were tailoring treatment decisions to individual patients [106]. In follow-up research, the authors found that higher statin-use rates for AMI patients with complex comorbidities were associated with both higher survival rates and higher adverse event rates [58]. Another study assessing the use of AA for new ischemic strokes found similar rates of AA use for patients with and without chronic kidney disease (CKD). In that study, higher AA treatment rates were only associated with better survival for stroke patients without CKD [59].

The results from our IV models suggest a tradeoff between survival and adverse events with increased use of BB+AA+ST. It is important to note that these estimates are only generalizable to marginal patients, i.e., those whose treatment choice were sensitive to the values of the instrument. The Local Average Treatment Effect estimate identified by IV estimators reflects the expected treatment effect for the “compliers,” or those whose treatment choice are conditional on the value of the specified instrumental variable(s). As such, the IV estimates found here will likely not generalize to the subset of patients who would have been offered the same medication combination by all providers. ATR-based instruments are particularly well-suited for this study’s research question, as parameter estimates are directly interpreted as the absolute change in outcome rates observed with higher rates of guideline-recommended therapy. In addition, because the treatment effects for these drug combinations are likely heterogeneous across patients, the optimal management strategy for any single individual cannot be directly addressed with these estimates.

Further limitations to this work should be acknowledged. As with any observational study, assumptions are required in regard to unmeasured confounders. In this study, hospital medical records were abstracted to assess the assumptions underlying the IV estimator. Although these measures were selected intentionally for this purpose and based upon evidence from the literature and expert input, they are likely not exhaustive of all factors related to treatment choice or patient outcomes. In addition, though no correlation was found between these instruments and the unmeasured confounders, only a finite set were measured.

The estimated treatment effects will be biased if other unmeasured and effective treatments are correlated with the study drugs. In McClellan’s seminal instrumental variable study, the authors acknowledged that their estimated effects of invasive surgical treatment on survival for Medicare AMI patients were likely biased high, as hospitals using invasive surgical treatment at higher rates were likely also using unmeasured treatments such as thrombolytics (at the time) at a higher rate [71]. This may very well be true for this study, as areas of the country which use the guideline-recommended drug combination (BB+AA+ST) at higher rates are also likely to be areas which promote the use of other medications such as aspirin, and which stress patient adherence to prescribed medications. As a result, the IV estimates reported here likely represent the upper bounds of the true impact of using the guideline-recommended treatment (BB+AA+ST) at higher rates relative to the alternative drug combinations.

Another limitation is the definition of treatment. The study medications are indicated for the management of a chronic condition, and prescribed to be taken indefinitely (as duration of adherence is correlated with outcomes) [13,29,109,110]. If patients fill a single prescription and do not use the full supply, or fail to fill subsequent prescriptions, then these models may not be informative regarding the full effects of (adherent) treatment. As such, this study cannot make inferences on differences in patient compliance across medication combinations.

In addition, adverse events were defined in this study as the presence of an inpatient claim listing any of the following diagnoses: hypotension, bradycardia, heart block, angioedema, hyperkalemia, serious myopathy, an acute renal event, or an acute hepatic event. These conditions were identified using medical coding terminology, and, although the observation period was limited to the first 90 days post-discharge, some encounters may not have been the direct result of treatment. Additionally, less severe events; other types of adverse events; and symptoms of adverse events reported by patients, but not indicated on inpatient claims, were not included in these measures. Thus, the true prevalence of adverse events is likely under-estimated. Given this, the results of this study likely represent a lower-bound of the true adverse-event rate in this population.

Lastly, this study utilized older Medicare data, and the treatment rates reported here may not reflect current practice. There is, however, evidence that the use of secondary-prevention medications for AMI patients has increased only moderately over time, even in cardiology practices [111]. Recent studies of Medicare beneficiaries [112,113,114], the Medical Expenditure Panel Survey (MEPS) [115], a registry cohort linked with Medicare claims [116,117,118], commercial insurance [119], and clinical trial [120] data report utilization rates similar to those found in this study (60–65% for ST and 35–40% for BB+AA+ST within 30 days post-discharge) and/or fairly stable treatment rates over the last 15 years. The patient mix within each drug combination, however, may have changed since the study period.

Even with these limitations, this present work is a contribution to the literature. Existing research evaluating the comparative effectiveness of drug combinations after AMI have applied risk-adjustment regression methods to observational data, and found more-intense therapy associated with better health outcomes [16,18,23,47,121,122,123]. However, only positive outcomes were assessed in these papers. Our study included both the potential benefits and harms associated with treatment. In addition, previous estimates are unbiased only under the assumption that all confounding factors related to treatment choice and outcomes were measured for each patient and controlled for in the analyses [49,51]. This is unlikely the case, given that the unmeasured confounders abstracted from the medical records in this study (and not available in other studies) were significantly associated with the treatment choice. The very use of the medical records data to evaluate model assumptions is another strength of this study.

## 5. Conclusions

This retrospective observational study assessed the benefits and detriments of combination therapy in a sample of Medicare beneficiaries hospitalized for AMI relative to guideline care (BB+AA+ST). The results from the instrumental variables (IV) models indicated tradeoffs between survival and adverse events associated with higher rates of guideline care relative to other drug combinations. This suggests that the observed distributions of drug combinations in practice may reflect a balance in treatment benefit and harm for the patient mix within each treatment group. These findings support the notion that decisions to deviate from guideline-recommended therapy do not necessarily signal poor quality-of-care, particularly in the context of polypharmacy for older, vulnerable adults [124].

## Figures and Tables

**Table 1:** Select Patient Characteristics and Outcomes of Medicare Beneficiaries Hospitalized with AMI by 30-day Post-discharge Drug Combination.

	Total	None	BB	AA	ST	BB+AA	BB+ST	AA+ST	BB+AA+ST
Sample size (N)	124,695	16,807	12,104	4790	4768	15,357	20,142	6187	44,540
Percent of full sample *		13.5%	9.7%	3.8%	3.8%	12.3%	16.2%	5.0%	35.7%
Female	57.3%	60.3%	58.8%	63.1%	53.9%	62.5%	53.5%	57.5%	55.3%
Median age (years)	78	81	80	80	78	79	77	77	76
Age (years)									
66–70	20.4%	14.0%	15.8%	15.7%	21.1%	18.2%	22.5%	20.2%	24.2%
71–75	19.9%	14.8%	18.0%	17.6%	20.9%	18.3%	21.3%	20.9%	22.4%
76–80	20.5%	18.7%	19.4%	19.7%	20.6%	19.5%	21.3%	22.5%	21.2%
81–85	18.6%	21.2%	19.1%	19.5%	18.9%	19.3%	17.9%	18.9%	17.5%
86+	20.6%	31.3%	27.6%	27.4%	18.5%	24.8%	17.1%	17.6%	14.7%
Race									
White	83.1%	81.8%	84.4%	80.5%	84.1%	83.8%	84.8%	81.4%	82.8%
Black	7.9%	10.3%	7.5%	9.5%	7.3%	8.0%	6.9%	7.8%	7.3%
Hispanic	5.9%	5.2%	5.4%	7.0%	5.7%	5.7%	5.2%	6.8%	6.5%
Other	3.1%	2.7%	2.7%	3.1%	2.9%	2.5%	3.1%	4.0%	3.4%
Number of CC									
0	34.5%	28.3%	29.6%	26.2%	30.6%	31.5%	38.3%	29.9%	39.5%
1	23.3%	21.5%	21.9%	25.0%	23.0%	24.4%	21.1%	25.5%	24.5%
2	14.2%	15.6%	14.7%	15.6%	13.9%	15.4%	13.4%	16.0%	13.2%
3+	28.0%	34.6%	33.8%	33.1%	32.5%	28.7%	27.2%	28.5%	22.8%
Chronic condition **									
IHD	56.5%	57.7%	60.4%	60.2%	60.8%	59.2%	55.9%	59.6%	53.1%
Heart failure	30.6%	39.5%	35.8%	39.1%	32.6%	35.6%	26.8%	31.4%	24.6%
Comp hypertensn	6.3%	6.5%	6.9%	7.6%	6.6%	6.9%	5.5%	7.4%	5.8%
unComp hypertensn	81.5%	80.4%	81.7%	88.0%	78.8%	86.0%	77.8%	85.9%	81.0%
Hyperlipidemia	66.9%	55.8%	61.9%	62.2%	73.4%	63.7%	70.4%	74.9%	70.7%
Diabetes mellitus	36.9%	37.4%	35.2%	40.5%	36.9%	39.2%	33.4%	41.3%	37.0%
COPD	25.9%	31.7%	29.0%	33.3%	33.4%	25.6%	24.7%	31.5%	21.2%
Atrial fibrillation	13.0%	16.4%	16.9%	17.0%	14.8%	15.0%	11.5%	13.4%	9.8%
Unstable angina	9.2%	8.6%	9.7%	9.7%	10.6%	9.4%	9.7%	9.9%	8.6%
NSTEMI	73.6%	77.7%	78.6%	78.8%	77.1%	74.9%	74.3%	75.5%	68.8%
Median LOS	6	7	6	6	6	6	6	6	5
Part D insurance									
Dual eligible	33.7%	43.2%	30.9%	36.1%	32.7%	32.1%	30.5%	35.9%	32.4%
Low-income subsidy	6.2%	5.5%	5.9%	5.9%	6.2%	6.5%	6.1%	6.2%	6.4%
One-year outcomes									
Overall survival	84.3%	71.4%	78.9%	79.1%	83.7%	82.8%	87.3%	86.2%	90.1%
CVE-free survival	75.4%	64.0%	70.5%	69.8%	75.4%	73.2%	78.7%	76.2%	80.8%
90-day outcome									
Adverse events	5.4%	6.7%	5.7%	6.6%	5.6%	5.9%	4.9%	5.3%	4.7%

Abbreviations: AMI = acute myocardial infarction; BB = beta-blocker; AA = ACE/ARB; ST = statin; CC = Charlson comorbidity; IHD = history of ischemic heart disease; Comp = complicated; hypertensn = hypertension; unComp = uncomplicated; COPD = chronic obstructive pulmonary disease; NSTEMI = non-ST segment elevation myocardial infarction; LOS = length of stay for initial acute hospitalization measured in days; CVE = cardiovascular event. * The denominator for each percent value reported in this row is the full sample. For all other percentages reported in the table, the denominator includes those with that drug combination (i.e., column percent). ** Chronic conditions were measured in the year prior to AMI and are not mutually exclusive.

**Table 2:** Mean Treatment Rate for Local Areas Grouped by Quintiles of ATR Values for Each Drug Combination, Percent Difference in Mean Treatment Rates Between the Highest and Lowest Quintile, and First-stage F-statistics of These Quintiles as Instruments in the IV Models.

Treatment Group	Full Sample	Areas in Q1 (Lowest Use)	Areas in Q2	Areas in Q3	Areas in Q4	Areas in Q5 (Highest Use)	%Δ	F-Statistic *
None	13.5%	9.7%	12.2%	13.4%	14.7%	17.4%	80	30.5 (*p* < 0.001)
BB	9.7%	6.1%	8.3%	9.6%	11.0%	13.6%	124	29.9 (*p* < 0.001)
AA	3.8%	1.6%	2.9%	3.8%	4.6%	6.3%	289	29.0 (*p* < 0.001)
ST	3.8%	1.6%	2.8%	3.9%	4.4%	6.4%	296	29.6 (*p* < 0.001)
BB+AA	12.3%	8.0%	10.6%	11.9%	14.2%	16.8%	110	34.5 (*p* < 0.001)
BB+ST	16.2%	11.4%	14.2%	16.1%	17.9%	21.2%	86	32.4 (*p* < 0.001)
AA+ST	5.0%	2.4%	3.9%	4.9%	6.1%	7.6%	220	29.7 (*p* < 0.001)
BB+AA+ST	35.7%	27.7%	32.9%	36.4%	38.9%	42.8%	54	reference group

Abbreviations: ATR = area treatment ratio; IV = instrumental variables; BB = beta-blocker; AA = ACE/ARB; ST = statin; Q = quintile; %Δ = percent difference in treatment rate between highest and lowest quintile; *p* = *p*-value. * F-statistics testing the exclusion restrictions on the instruments (ATR-based quintiles) from the first-stage equations of the IV models. This test is used to assess the instruments in jointly predicting treatment choice. Insignificant test statistics or significant tests with values less than 10 indicate weak instruments.

**Table 3:** Parameter Estimates from IV Models of the Comparative Effectiveness of Various Post-discharge Drug Combinations on Positive and Negative Outcomes Relative to Guideline-Recommended Combination Therapy *.

	One-Year Overall Survival			One-Year CVE-Free Survival			90-Day Adverse Events		
Treatment Group	β Coef	SE	*p*-Value	β Coef	SE	*p*-Value	β Coef	SE	*p*-Value
None	−12.91	3.63	<0.001	−6.60	4.37	0.130	−5.61	2.42	0.020
BB	−8.66	3.90	0.026	−8.18	4.68	0.080	−3.74	2.62	0.153
AA	−8.23	6.17	0.182	−6.30	7.40	0.395	−0.45	4.06	0.911
ST	−2.41	5.99	0.687	0.89	7.21	0.901	−9.15	3.97	0.021
BB+AA	−9.46	3.41	0.006	−7.81	4.09	0.056	−6.10	2.28	0.007
BB+ST	−3.93	3.20	0.220	−4.33	3.86	0.261	−0.22	2.15	0.919
AA+ST	−1.91	5.52	0.729	−4.81	6.63	0.469	0.01	3.66	0.999

Abbreviations: IV = instrumental variables; CVE = cardiovascular event; β coef = beta coefficient (parameter estimate); SE = robust standard error; BB = beta-blocker; AA = ACE/ARB; ST = statin; CVE = cardiovascular event. * Guideline-recommended combination therapy (BB+AA+ST) was set as the reference group. Parameter estimates reflect absolute effects, and are measured in percentage points. Mean (full sample) outcomes were 84.3% for overall survival, 75.4% for CVE-free survival, and 5.4% for adverse events. Covariates included in the models are listed in File S1.

**Table 4:** Variables Unavailable in Medicare Claims Created from Abstracted Medical Records (N = 1404) for Patients Grouped by Post-discharge Drug Combination *.

	Full Sample	None	BB	AA	ST	BB+AA	BB+ST	AA+ST	BB+AA+ST	*p*-Value **
Severity of AMI	7.55	6.77	7.03	6.76	7.96	7.21	8.01	8.17	7.89	<0.001
Disease burden	3.37	2.99	3.16	2.99	3.83	3.13	3.49	3.60	3.53	<0.001
% w/potential contraindication	46.30	54.84	52.03	57.04	52.14	48.97	43.72	43.29	34.00	<0.001
ADL	0.43	0.91	0.66	0.56	0.48	0.40	0.27	0.27	0.28	<0.001
% w/diff in any ADL domain	25.71	45.16	34.46	29.63	25.71	26.80	17.59	19.51	19.67	<0.001
% w/diff in 2+ ADL domains	7.05	14.52	10.14	9.63	7.14	6.19	4.52	3.05	5.67	<0.001
ACE-27 score	1.87	2.04	2.03	2.03	1.96	1.89	1.75	1.87	1.67	<0.001
% overweight (BMI > 25)	66.95	51.61	61.49	62.22	70.00	66.49	66.33	73.17	74.00	<0.001
% underweight (BMI < 18.5)	3.70	8.06	4.05	5.19	6.43	3.09	3.52	1.83	1.33	<0.001
% cath w/in 24 h	39.32	16.94	27.70	23.70	38.57	30.41	52.76	45.12	55.33	<0.001

Abbreviations: BB = beta-blocker; AA = ACE/ARB; ST = statin; AMI = acute myocardial infarction; diff = difficulty; ADL = activities of daily living; ACE-27 = adult comorbidity evalaluation−27; % = percent; w/ = with; BMI = body mass index; % cath w/in 24 h = percent undergoing cardiac catheterization within 24 h of admission. * Description and definition of these variables are detailed in File S3. ** Ordinary least squares model used to test for linear trend.

**Table 5:** Variables Unavailable in Medicare Claims Created From Abstracted Medical Records (N = 1404) for Patients Grouped by the Instrument (Quintiles for ATR Values) for Guideline-concordant Treatment (BB+AA+ST) *.

	Full Sample	Areas in Quintile 1 (Lowest Use)	Areas in Quintile 2	Areas in Quintile 3	Areas in Quintile 4	Areas in Quintile 5 (Highest Use)	*p*-Value **
Severity of AMI	7.55	7.43	7.88	7.62	7.44	7.40	0.589
Disease burden	3.37	3.46	3.35	3.33	3.24	3.46	0.700
% w/ potential contraindication	46.30	44.26	44.52	47.39	47.4	48.19	0.255
ADL	0.43	0.48	0.37	0.46	0.42	0.43	0.840
% w/diff in any ADL domain	25.71	25.00	23.32	28.92	25.61	25.70	0.643
% w/diff in 2+ ADL domains	7.05	7.77	6.71	6.27	7.27	7.23	0.894
ACE-27 score	1.87	1.93	1.83	1.89	1.85	1.84	0.403
% overweight (BMI > 25)	66.95	66.22	68.55	66.2	67.82	65.86	0.901
% underweight (BMI < 18.5)	3.70	2.36	4.24	4.53	4.50	2.81	0.667
% cath w/in 48 h	39.32	41.28	38.79	39.86	36.65	40.00	0.610

Abbreviations: BB = beta-blocker; AA = ACE/ARB; ST = statin; AMI = acute myocardial infarction; diff = difficulty; ADL = activities of daily living; ACE-27 = adult comorbidity evalaluation−27; % = percent; w/ = with; BMI = body mass index; % cath w/in 24 h = percent undergoing cardiac catheterization within 24 h of admission. * Description and definition of these variables are detailed in File S3. ** Ordinary least squares model used to test for linear trend.

## Data Availability

Restrictions apply to the availability of these data. Medicare data were obtained from the Research Data Assistance Center (ResDAC), a Centers for Medicare and Medicaid Services (CMS) contractor. These data are available from ResDAC at https://resdac.org/research-identifiable-files-rif-requests (accessed on 31 October 2022) with the permission of CMS. The acquisition and abstraction of hospital medical records data was conducted by Information College Enterprises (ICE) with permission from, and in collaboration with, CMS and Buccaneer.

## Supplementary Materials

The following supporting information can be downloaded at: https://www.mdpi.com/article/10.3390/pharmacy10060147/s1, File S1: Claims-based Covariates Included in All Instrumental Variables (IV) Models; File S2: Creation of ATR Instruments and Model Specification for IV Analyses; File S3. Creation of Unmeasured Confounders from Abstracted Medical Records; Table S1: Definitions of variables created from abstracted hospital medical records; Table S2. Characteristics of a stratified, random subsample of the study cohort (N = 1404) for variables of “unmeasured confounders” created using abstracted medical records data; Table S3. Percent of sample with individual indicators for measures of severity of AMI, disease burden, and potential contraindications. References [125,126,127,128,129,130] are cited in the supplementary materials.
