# Supplementary material for: Generating Practice-Based Evidence in the Use of Guideline-Recommended Combination Therapy for Secondary Prevention of Acute Myocardial Infarction
Source: Pharmacy (Basel). 2022 Nov 3;10(6):147. doi: 10.3390/pharmacy10060147 (PMC9680510; doi:10.3390/pharmacy10060147)
Supplement: Supplementary file 1 [file pharmacy-10-00147-s001.zip › pharmacy-1927891-supplementary.pdf]

File S1. Claims-based Covariates Included in All Instrumental Variables (IV) Models

Demographic variables: age group in years (66-70, 71-75, 76-80, 81-85, 85+), race/ethnicity (White, Native American, Asian, Black, Hispanic, Race other, Race unknown), and sex.

Primary AMI diagnosis for initial acute hospitalization: anterior wall, subendocardial infarction, and other location.

Baseline medical history and comorbidity (two sets of variables, measured one-year prior to index admission date and during AMI hospitalization): number of Charlson comorbidity, unstable angina, cardiac arrest, ventricular arrhythmia, other cardiac arrhythmia, atrial fibrillation, stroke, ischemic heart disease, heart failure, complicated hypertension, uncomplicated hypertension, metastatic neoplasm, other neoplasm, and transient ischemic attack.

Procedures (two sets of variables unless specified, measured one-year prior to index admission date and during AMI hospitalization): coronary artery bypass grafting (CABG), coronary stent, pacemaker implantation, ventricular assist device (VAD), percutaneous transluminal coronary angioplasty (PTCA), cardiac catherization (during AMI hospitalization), echocardiogram (during AMI hospitalization), and stress test (during AMI hospitalization).

Medication use (180-days prior to index admission date): ACE/ARB, beta-blocker, statin, calcium-channel blocker, clopidogrel, diuretic (loop, thiazide, potassium sparing), anti-diabetic (e.g. alpha-glucosidase, amylin analogue, biguanide, dipeptidyl peptidase 4 inhibitors, glucagon-like peptide 1 receptor agonist, insulin, meglitinide, sulfonylurea, thiazolidinedione, epalrestat, exenatide, glybuzole), low molecular weight heparin, nitrate, other antihypertensive, fenofibrate and other lipid lowering agents, and warfarin.

Evidence of potential contraindication to study drugs (two sets of variables unless specified, measured one-year prior to index admission date and during AMI hospitalization): angioedema, hyperkalemia, renal event, disorders of lipoid metabolism, hypotension, bradycardia, heart block, cardiogenic shock (during AMI hospitalization), nonserious myopathy, serious myopathy, hepatic event, chronic kidney disease, depression, diabetes, chronic obstructive pulmonary disease, and asthma.

Healthcare utilization variables (during AMI hospitalization): days in intensive care unit (ICU), days in cardiac/coronary care unit (CCU), days in intermediate care unit (IMC), days in other acute-care facility, days in other non-acute-care facility, ER use, and transfer to another facility.

Insurance variables and measure of financial burden: low-income subsidy (LIS), dually-eligible for both Medicare and Medicaid benefits at time of discharge, changed dual-eligibility in year of AMI hospitalization, Part D benefit phase at time of discharge, Part D plan premium net rebate amount in quartiles, cumulative drug costs (from beginning of year to index admission date) in deciles, cumulative amount beneficiary paid out-of-pocket for prescription medications (from beginning of year to index admission date) in quartiles.

Urban and socioeconomic (SES) variables (measured at the census-tract-level of the beneficiary's residence): rural-urban commuting area (RUCA) code (metropolitan area, non-metropolitan area, RUCA unknown), above-median in percent immigrant, above-median in percent with non-English speakers, above-median in percent with low income, above-median in percent graduating from high school, and above-median in percent living in poverty.

File S2. Creation of ATR Instruments and Model Specification for IV Analyses

The idea of Instrumental Variables (IV) analyses as a solution to the problem of identification (e.g. selection bias) was first noted over a hundred years ago,[1] and has been used in health services research over the last three decades.[2-11] The intuition behind this approach is similar to that used in randomized controlled trials (RCTs): patients are randomized into groups by "instruments," variables that affect treatment choice but have no direct effect on outcomes (except to the extent that they influence the choice of treatment) and then outcomes are compared across the instruments to estimate the effect of treatment.[12-14]

This study builds on prior research developing instrumental variables based on geographic variation in practice styles.[5,9,10,15-31] Area Treatment Ratios (ATRs), a measure of an area's propensity towards a specific treatment relative to other areas, were used in this study as the instrumental variables. These were created following the approach developed by Fang et al.[32] First, "local areas" are identified for each patient based on the driving area for clinical care (DACC) method which gathers a threshold numbers of closest residing patients (at the level of the patient's residence ZIP code).[7,33] A threshold of 150 patients was set in this study with sensitivity analyses conducted using thresholds of 50, 100 and 200 patients. Every patient is then assigned an ATR value, one for each of the eight drug combinations and calculated as the ratio of the actual treatment rate within the patient's local area to the predicted rate (estimated with the full sample and controlling for measured patient- and area-level characteristics). By definition, ATRs are a continuous measure, strictly positive and distributed around one.[22] The ATRs are then grouped into quintiles and transformed into binary variables. These indicator variables form the set of instruments used in the IV analyses. For illustrative purposes, ATR values were mapped for the northeast portion of the United States for four of the eight drug combinations (Figure 1).

For the IV analyses, a two-stage least squares (2SLS) estimator was specified. Eight treatment-choice equations were modeled in the first stage, one for each drug combination (Equation 1).

$$DC_{ij} = \beta_0 + \sum_{j=1}^{8}\sum_{k=1}^{5} \beta_{kj} qATR_{kji} + \vec{X}_i \beta + \epsilon_i \quad (1)$$

In this equation, X_i includes all measured covariates (File S1) for patient i and $qATR_{kj}$ corresponds to the a set of binary, indicator variables representing whether patient i resided in a local area grouped into quintile k of ATR values for drug combination DC_j. Although all five quintiles were listed for all eight drug combinations in the first-stage equation, the ATR quintiles for guideline-recommended treatment (three drug combination, BB+AA+ST) and the first quintile (lowest-use areas) for the remaining ATRs were dropped from the model and set as the reference groups.

The second-stage equation for outcome Y (Equation 2) included the same set of measured covariates X_i along with the predicted probability of patient i receiving drug combination $\widehat{DC}_n$ (calculated from first-stage parameter estimates). Again, guideline-recommended treatment (three drug combination, BB+AA+ST) was set as the reference case and dropped when estimating the model.

$$Y_i = \beta_0 + \sum_{n=1}^{8} \beta_n \widehat{DC_{ni}} + \vec{X}_i \beta + \eta_i \quad (2)$$

File S3. Creation of Unmeasured Confounders From Abstracted Medical Records

In this study, additional data were obtained to test the assumptions underlying the IV model. Medical records from the initial AMI hospitalization were abstracted for a subset of the study cohort to create variables of known confounders[34-37] that are typically unmeasured in observational, comparative effectiveness studies using administrative data because the information is not directly available in the medical claims. Data elements were abstracted to create "unmeasured confounders" and merged with the claims-based analytical dataset. Details regarding design of the cohort-selection algorithm, CMS-approved process of re-identifying patients and acquisition of medical records, and development and used of a data abstraction tool are found elsewhere.[38]

In summary, a stratified random sample was identified, stratified by residence in the fifth (highest) quintile in one of eight ATRs and eight observed drug combinations, and balanced across the four U.S. Census Geographical Regions (i.e., a total of 64 primary sampling units - from which the sample was selected). Hospital medical records were obtained from an initial sample of 1,920 requested records. To ensure that data were abstracted accurately and uniformly across all members of the abstraction team, results from all three internal quality control rounds of evaluation were aggregated by domain (e.g. administrative variables, lab values, etc). Because of the voluntary nature of the record request, patient characteristics were compared for whom records were and were not received, using Medicare A and B claims from the index hospitalization. Key dimensions that were compared included age, gender, comorbidity history, complications during the stay, and length of stay. Very few differences were found.[38] Abstracted medical records tended to belong to patients who were slightly older, had shorter average acute care lengths of stay, were less likely to require an acute care transfer, and were more likely to be discharged home than another facility. Records from facilities in the northeast region of the U.S. and larger facilities (300+ beds) were less likely to be received.

A structured data abstraction tool, based on one developed by the Cooperative Cardiovascular Project, was created to obtain information from the medical records of the sampled patients for the index hospital stay, which could have included treatment at two facilities if the patient was transferred during the acute stay. Variables were modified and customized in consultation with study team cardiologists, internists, and nurses. The domains of information and examples of the types of data elements captured by the medical record abstraction tool included patient clinical information (e.g. body mass index [BMI], smoking status), AMI diagnosis at admission, presenting symptoms, initial vital signs and lab tests, prior history (e.g. drug allergies, conditions, procedures, hospitalizations, and status immediately prior to admission), medication use prior to admission and during the hospital stay, in-hospital procedures or complications, and in-hospital labs and test results collected prior to discharge.

Variables created from these data were developed specifically to reflect risk of second AMI (i.e. potential benefit from treatment) as well as risk of treatment-related adverse events (i.e. potential harm from treatment). Some variables were created from existing algorithms and others were developed by the study team (measures termed "severity of AMI" and "disease burden"). Even though the latter are not validated in terms of their prognostic value, they are unable to be measured in claims data and should be (are assumed to be) randomly distributed across the treatment/instruments of the statistical models.

Table S1. Definitions of variables created from abstracted hospital medical records

1. Severity of AMI defined as the sum of the following items:
Complications During Stay
Pulmonary edema
Hypotension
Bradycardia
Cardiogenic shock
Acute heart failure
Another acute myocardial infarction (AMI)
Resuscitated cardiac arrest
Lab Tests During Stay
Highest recorded troponin level >1.0

Procedures/Interventions During Stay

- Ejection fraction (EF) less than 35% during first/only coronary angiogram
- More than 1 coronary angiogram
- Coronary artery bypass graft (CABG)
- Cardiac catheterization within 6 hours of admission
- Cardiac resynchronization therapy
- Ventricular assist device
- Intra-aortic balloon pump
- Positive airway pressure (PAP) treatment
- Use of ventilator
- Hemodialysis
- Heart transplant
- Implantable cardioverter defibrillator (ICD) placed within 24 hours of admission

Initial Vitals/Tests During Stay

- Initial systolic blood pressure (at admission) <100

Diagnostic Tests During Stay

- Results from Left Ventricle Function (LVF) assessment recorded from cardiac catheterization
- Echocardiogram within 6 hours of admission
- Akinesia, cardiac aneurysm, cardiac valvular disease, cardiomyopathy, dyskinesia, heart failure, pericardial effusion or tamponade, or pulmonary hypertension found on Echocardiogram
- Nuclear imaging performed
- Multigated acquisition (MUGA) scan performed
- Positron emission tomography (PET) scan performed
- Findings from electrocardiogram (ECG) closest to discharge: New Q-wave
- Findings from ECG closest to discharge: ST-elevation
- Findings from ECG closest to discharge: New left bundle branch block, unspecified time
- Findings from ECG closest to discharge: Ventricular tachycardia
- Findings from ECG closest to discharge: Accelerated idioventricular rhythm
- Findings from ECG closest to discharge: Ventricular fibrillation
- Findings from ECG closest to discharge: Atrioventricular block
- Findings from ECG closest to discharge: First-degree heart block
- Findings from ECG closest to discharge: Second-degree heart block
- Findings from ECG closest to discharge: Second-degree heart block, Type 1
- Findings from ECG closest to discharge: Second-degree atrioventricular block: Mobitz I or Wenckebach (Type 1)
- Findings from ECG closest to discharge: Type 2 Second-degree heart block
- Findings from ECG closest to discharge: Second-degree atrioventricular block: Mobitz II (Type 2)
- Findings from ECG closest to discharge: Third-degree heart block
- Chest x-ray findings: Acute pulmonary edema
- Chest x-ray findings: Pulmonary vasculature engorgement
- Chest x-ray findings: Enlarging heart

2. Disease burden defined as the sum of the following items:

- Patient Characteristics (at time of admission)
  - Current smoker
  - BMI ≥38
- Prior Medical Conditions
  - History of angina
  - History of hypertension
  - History of peripheral artery disease (PAD)
  - History of limb amputation due to PAD
  - History of diabetes mellitus
  - History of severe carotid artery stenosis
  - History of deep vein thrombosis (DVT)
  - History of stroke or Transient Ischemic Attack (TIA)
  - Timing of stroke/TIA was recent
  - Severity of stroke/transient ischemic attack (TIA): significant deficit
- Prior Procedures/Interventions
  - History of PAD revascularization
  - History of percutaneous transluminal coronary angioplasty (PTCA)
  - History of carotid endarterectomy
  - History of aortic aneurysm repair
- Complications During Stay
  - Abdominal aortic aneurysm during the stay
  - Renal artery stenosis during the stay
  - Lower extremity stenosis >50% during the stay
  - Carotid artery stenosis >50% during the stay
  - Deep vein thrombosis (DVT) during the stay
- Procedures/Interventions During Stay
  - Stent placed during stay
  - Resuscitated cardiac arrest during the stay
- Lab Results For Those Tested During Stay
  - Total cholesterol >200, out-of-range (high)
  - Triglycerides >200, out-of-range (high)
  - Low density lipoproteins (LDL) >100, out-of-range (high)
  - High density lipoproteins (HDL) <40, out-of-range (low)

3. Potential contraindication to study drugs if any of the following:

- History of chronic obstructive pulmonary disease (COPD) with dyspnea
- Pulmonary embolism during hospitalization
- Pulmonary hypertension found on echocardiogram during hospitalization
- Findings from ECG closest to discharge: Third-degree heart block
- Renal arterial stenosis as a complication during hospitalization

History of chronic kidney disease<br>Acute renal failure during hospitalization for AMI<br>History of moderate/severe aortic stenosis<br>History of rhabdomyolysis<br>History of statin-related muscle symptoms<br>Statin-related muscle symptoms during hospitalization
4. Difficulty with activities of daily living (ADLs) defined as the number of domains in which the patient is dependent on others (i.e. requires supervision, direction, personal assistance, or total care) per the Katz et al.[39] Larger numbers reflect greater dependence and need for assistance in activities of daily living.
4a. Whether the patient was dependent on others for any ADL domain.
4b. Whether the patient was dependent on others for 2 or more ADL domains.
5. Adult Comorbidity Evaluation (ACE)-27 score based on Piccirillo et al. and calculated with the algorithm provided by Washington University School of Medicine's Clinical Outcomes Research Office.[40,41]
6. Overweight (BMI ≥25)
7. Underweight (BMI <18.5)
8. Cardiac catheterization within 24 hours of admission

Descriptive statistics of these "unmeasured confounders" are reported in eTable 2 with the distribution of individual indicators for the variables listed in eTable 3. The majority (67.0%) were overweight and 39.3% underwent cardiac catheterization within 24 hours of admission. Severity of AMI was proxied with a single measure as the summation of 44 individual indicators. Only 3.0% of the sample had none of these indicators documented in the medical records. Half (52.2%) had 6 or fewer of the indicators and 10.7% had 14 or more indicators documented. With regards to specific indicators, most of the sample (65.0%) had a documented troponin level >1.0 at some point in the stay, 7.6% had an ejection fraction <35% on the first/only coronary angiogram, 7.3% had an initial (admission) systolic blood pressure reading <100, and 2.1% experienced a resuscitated cardiac arrest during the stay.

"Disease burden" was a variable created to proxy severity of cardiovascular disease and included a number of patient characteristics associated with treatment and outcomes not measurable in claims data. As an overall measure, three-quarters of patients (75.7%) had four or fewer of the indicators. With regards to the individual indicators, 13.0% had a BMI ≥38, 12.5%

were identified as current smokers, and 37.4% had an LDL >100 (out-of-range high). In this cohort, 83.8% had a documented history of hypertension and 36.4% had a documented history of diabetes mellitus, values very similar to those measured in the study cohort using Medicare claims (81.5% and 36.9%, respectively) in Table 1.

Table S2. Characteristics of a stratified, random subsample of the study cohort (N=1,404) for variables of "unmeasured confounders" created using abstracted medical records data

	mean	SD	Minimum	Maximum
Severity of AMI	7.55	4.49	0	32
Disease burden	3.67	1.82	0	12
Percent with potential contraindication*	46.30	49.88	0	100
ADL	0.43	1.02	0	6
Percent with diff in any ADL domain	25.71	43.72	0	100
Percent with diff in 2+ ADL domains	7.05	25.61	0	100
ACE-27 score	1.87	0.95	0	3
Percent overweight (BMI ≥25)	66.95	47.06	0	100
Percent underweight (BMI <18.5)	3.70	18.89	0	100
Percent cath w/in 24 hrs	39.32	48.86	0	100

Abbreviations: SD=standard deviation; AMI=acute myocardial infarction; ADL=activities of daily living; diff=difficulty; ACE-27=adult comorbidity evalaluation-27; BMI=body mass index.
* Percent with a potential contraindication to study drugs.

The ACE-27 score and ADLs were created to proxy performance/functional status and frailty. The mean ACE-27 score for the sample was 1.87 (where a 3 is "severe"). This "high" score reflects the number of cogent comorbid ailments of the cardiovascular system included in the index. In terms of activities of daily living (ADLs), 25.7% had difficulty in one or more (of the six) domains and 7.1% had difficulty with two or more domains. Most patients were considered overweight (67.0%) and a small minority were underweight (3.7%).

Almost half of the subsample (43.4%) had a potential contraindication to the study drugs and 17.9% had two or more of the 11 individual indicators. For those with a potential contraindication, the most common was history of chronic kidney disease (40.3%). Most (67.2%)

of those with chronic kidney insufficiency listed in the medical records did not require dialysis. However, 13.0% did require dialysis and 4.2% had a history of multi-organ failure, shock, or sepsis (with acute dialysis). Additional common potential contraindications included acute renal failure during the AMI hospitalization (35.5%), findings of pulmonary hypertension on echocardiogram (29.7%), history of COPD with dyspnea (36.9%), and history of statin-related muscular problems (1.5%).

Table S3. Percent of sample with individual indicators for measures of severity of AMI, disease burden, and potential contraindications

Severity of AMI	
Complications During Stay	
Pulmonary edema	12.5%
Hypotension	18.4%
Bradycardia	20.9%
Cardiogenic shock	2.6%
Acute heart failure	36.3%
Another acute myocardial infarction (AMI)	1.1%
Resuscitated cardiac arrest	2.1%
Lab Tests During Stay	
Highest recorded troponin level >1.0	65.0%
Procedures/Interventions During Stay	
EF less than 35% during first/only coronary angiogram	7.6%
More than 1 coronary angiogram	1.1%
Coronary artery bypass graft (CABG)	7.1%
Cardiac catheterization within 6 hours of admission	21.4%
Cardiac resynchronization therapy	0.0%
Ventricular assist device	--
Intra-aortic balloon pump	3.0%
Positive airway pressure (PAP) treatment	5.6%
Use of ventilator	4.2%
Hemodialysis	2.1%
Heart transplant	0.0%
Implantable cardioverter defibrillator (ICD) within 24 hours of admission	--
Initial Vitals/Tests During Stay	
Initial systolic blood pressure (admission) <100	7.3%
Diagnostic Tests During Stay	
Results from Left Ventricle Function (LVF) assessment recorded from cardiac catheterization	33.9%
Echocardiogram performed within 6 hours of admission	8.1%

Akinesia, cardiac aneurysm, cardiac valvular disease, cardiomyopathy, dyskinesia, heart failure, pericardial effusion or tamponade, or pulmonary hypertension found on Echocardiogram	57.5%
Nuclear imaging performed	6.2%
Multigated acquisition (MUGA) scan performed	--
Positron emission tomography (PET) scan performed	--
Findings from electrocardiogram (ECG) closest to discharge: New Q-wave	3.8%
Findings from ECG closest to discharge: ST-elevation	7.7%
Findings from ECG closest to discharge: New left bundle branch block, unspecified time	2.1%
Findings from ECG closest to discharge: Ventricular tachycardia	--
Findings from ECG closest to discharge: Accelerated idioventricular rhythm	0%
Findings from ECG closest to discharge: Ventricular fibrillation	--
Findings from ECG closest to discharge: Atrioventricular block	10.0%
Findings from ECG closest to discharge: First-degree heart block	--
Findings from ECG closest to discharge: Second-degree heart block	--
Findings from ECG closest to discharge: Second-degree heart block, Type 1	--
Findings from ECG closest to discharge: Second-degree atrioventricular block: Mobitz I or Wenckebach (Type 1)	--
Findings from ECG closest to discharge: Type 2 Second-degree heart block	--
Findings from ECG closest to discharge: Second-degree atrioventricular block: Mobitz II (Type 2)	0%
Findings from ECG closest to discharge: Third-degree heart block	--
Chest x-ray findings: Acute pulmonary edema	11.2%
Chest x-ray findings: Pulmonary vasculature engorgement	7.5%
Chest x-ray findings: Enlarging heart	5.1%
**Disease burden**	
Patient Characteristics (at time of admission)	
Current smoker	12.5%
BMI ≥38	13.0%
Prior Medical Conditions	
History of angina	48.1%
History of hypertension	83.8%
History of peripheral artery disease (PAD)	18.4%
History of limb amputation due to PAD	--
History of diabetes mellitus	33.0%
History of severe carotid artery stenosis	5.4%
History of deep vein thrombosis (DVT)	1.2%
History of stroke/transient ischemic attack (TA)	--
Timing of stroke/TIA was recent	1.3%
Severity of stroke/TIA: significant deficit	1.9%
Prior Procedures/Interventions	
History of PAD revascularization	3.1%
History of percutaneous transluminal coronary angioplasty (PTCA)	19.9%
History of carotid endarterectomy	5.4%

History of aortic aneurysm repair	2.5%
Complications During Stay	
Abdominal aortic aneurysm during the stay	0.8%
Renal artery stenosis during the stay	--
Lower extremity stenosis >50% during the stay	--
Carotid artery stenosis >50% during the stay	1.3%
Deep vein thrombosis (DVT) during the stay	1.7%
Procedures/Interventions During Stay	
Stent placed during stay	36.4%
Resuscitated cardiac arrest during the stay	2.1%
Lab Results For Those Tested During Stay	
Total cholesterol >200, out-of-range (high)	15.5%
Triglycerides >200, out-of-range (high)	21.8%
Low density lipoproteins (LDL) >100, out-of-range (high)	37.4%
High density lipoproteins (HDL) <40, out-of-range (low)	51.4%
Potential contraindication to study drugs	
History of chronic obstructive pulmonary disease (COPD) with dyspnea	17.1%
Pulmonary embolism during hospitalization	0.8%
Pulmonary hypertension found on echocardiogram during hospitalization	13.7%
Findings from ECG closest to discharge: Third-degree heart block	--
Renal arterial stenosis as a complication during hospitalization	--
History of chronic kidney disease	18.7%
Acute renal failure during hospitalization	16.5%
History of moderate/severe aortic stenosis	2.7%
History of rhabdomyolysis	--
History of statin-related muscle symptoms	--
Statin-related muscle symptoms during hospitalization	--

-- numbers suppressed due to small cell size (N<11).

References

1. Staiger, D.; Stock, J.H. Instrumental Variables Regression with Weak Instruments. *Econometrica* **1997**, *65*, 557, https://doi.org/10.2307/2171753.
2. Wright P.G. Moore's Economic Cycles. Q J Econ 1915, 29, 631-641. doi:10.2307/1885466
3. McClellan, M.; McNeil, B.J.; Newhouse, J.P. Does more intensive treatment of acute myocardial infarction in the elderly reduce mortality? Analysis using instrumental variables. *JAMA* **1994**, *272*, 859–66.
4. McClellan M, Newhouse JP. Instrumental Variables Analysis Applications in Health Services Research - a Special Supplement to Hsr - Overview of Supplement Issue. Health Serv Res. 2000;35(5):1061-1069.
5. Newhouse, J.P. Instrumental Variables in Health Services Research. Wiley StatsRef: Statistics Reference Online 2014, doi.org/10.1002/9781118445112.stat05330
6. Polgreen, L.A.; Cook, E.A.; Brooks, J.M.; Tang, Y.; Polgreen, P.M. Increased Statin Prescribing Does Not Lower Pneumonia Risk. *Clin. Infect. Dis.* **2015**, *60*, 1760–1766, https://doi.org/10.1093/cid/civ190.
7. Brooks JM, Chapman CG, Suneja M, et al. Use of Angiotensin-Converting Enzyme Inhibitors and Angiotensin Receptor Blockers for Geriatric Ischemic Stroke Patients: Are the Rates Right? J Am Heart Assoc. 2018;7(11).
8. Brooks JM, Cook E, Chapman CG, et al. Statin Use after Acute Myocardial Infarction by Patient Complexity: Are the Rates Right? Med Care. 2015;53(4):324-331.
9. Brooks JM, McClellan M, Wong HS. The Marginal Benefits of Invasive Treatments for Acute Myocardial Infarction: Does Insurance Coverage Matter? Inquiry. 2000;37(1):75-90.
10. Chapman, C.G.; Floyd, S.B.; Thigpen, C.A.; Tokish, J.M.; Chen, B.; Brooks, J.M. Treatment for Rotator Cuff Tear Is Influenced by Demographics and Characteristics of the Area Where Patients Live. *JBJS Open Access* **2018**, *3*, e0005, https://doi.org/10.2106/jbjs.oa.18.00005.
11. Floyd, S.B.; Campbell, J.; Chapman, C.G.; Thigpen, C.A.; Kissenberth, M.J.; Brooks, J.M. Geographic variation in the treatment of proximal humerus fracture: an update on surgery rates and treatment consensus. *J. Orthop. Surg. Res.* **2019**, *14*, 22, https://doi.org/10.1186/s13018-018-1052-2.
12. McDowell BD, Chapman CG, Smith BJ, Button AM, Chrischilles EA, Mezhir JJ. Pancreatectomy Predicts Improved Survival for Pancreatic Adenocarcinoma: Results of an Instrumental Variable Analysis. Ann Surg. 2015;261(4):740-745. doi:10.1097/sla.0000000000000796.
13. Angrist JD, Imbens GW, Rubin DB. Identification of Causal Effects Using Instrumental Variables. J Am Stat Assoc. 1996;91(434):444-455.
14. Angrist, J.D.; Krueger, A.B. Instrumental Variables and the Search for Identification: From Supply and Demand to Natural Experiments. *J. Econ. Perspect.* **2001**, *15*, 69–85, https://doi.org/10.1257/jep.15.4.69.
15. Greenland, S.; Morgenstern, H. Confounding in Health Research. *Annu. Rev. Public Heal.* **2001**, *22*, 189–212, https://doi.org/10.1146/annurev.publhealth.22.1.189.
16. Brooks, J.M.; Chrischilles, E.A. Heterogeneity and the Interpretation of Treatment Effect Estimates From Risk Adjustment and Instrumental Variable Methods. *Med Care* **2007**, *45*, S123–S130, https://doi.org/10.1097/mlr.0b013e318070c069.
17. Brooks, J.M, McClellan M, Wong HS. The Marginal Benefits of Invasive Treatments for Acute Myocardial Infarction: Does Insurance Coverage Matter? Inquiry-the Journal of Health Care Organization Provision and Financing. 2000;37(1):75-90.—重复
18. Brookhart, M.A.; Wang, P.S.; Solomon, D.H.; Schneeweiss, S. Evaluating Short-Term Drug Effects Using a Physician-Specific Prescribing Preference as an Instrumental Variable. *Epidemiology* **2006**, *17*, 268–275, https://doi.org/10.1097/01.ede.0000193606.58671.c5.
19. Rassen, J.A.; Brookhart, M.A.; Glynn, R.J.; Mittleman, M.A.; Schneeweiss, S. Instrumental variables II: instrumental variable application—in 25 variations, the physician prescribing preference generally was strong and reduced covariate imbalance. *J. Clin. Epidemiology* **2009**, *62*, 1233–1241, https://doi.org/10.1016/j.jclinepi.2008.12.006.
20. Stukel TA, Fisher ES, Wennberg DE, Alter DA, Gottlieb DJ, Vermeulen MJ. Analysis of Observational Studies in the Presence of Treatment Selection Bias: Effects of Invasive Cardiac Management on Ami Survival Using Propensity Score and Instrumental Variable Methods. JAMA. 2007;297(3):278-285.
21. Brooks, J.M.; Chrischilles, E.A.; Scott, S.D.; Chen-Hardee, S.S. Was Breast Conserving Surgery Underutilized for Early Stage Breast Cancer? Instrumental Variables Evidence for Stage II Patients from Iowa. *Heal. Serv. Res.* **2003**, *38*, 1385–1402, https://doi.org/10.1111/j.1475-6773.2003.00184.x.

22. Fang, G.; Brooks, J.M.; Chrischilles, E.A. Comparison of Instrumental Variable Analysis Using a New Instrument With Risk Adjustment Methods to Reduce Confounding by Indication. *Am. J. Epidemiology* **2012**, *175*, 1142–1151, https://doi.org/10.1093/aje/kwr448.
23. Schroeder, M.C.; Tien, Y.-Y.; Wright, K.; Halfdanarson, T.R.; Abu-Hejleh, T.; Brooks, J.M. Geographic variation in the use of adjuvant therapy among elderly patients with resected non-small cell lung cancer. *Lung Cancer* **2016**, *95*, 28–34, https://doi.org/10.1016/j.lungcan.2016.02.010.
24. Tang, Y.; Brooks, J.M.; Wetmore, J.B.; Shireman, T.I. Association between higher rates of cardioprotective drug use and survival in patients on dialysis. *Res. Soc. Adm. Pharm.* **2015**, *11*, 824–843, https://doi.org/10.1016/j.sapharm.2014.12.007.
25. Polgreen, L.A.; Cook, E.A.; Brooks, J.M.; Tang, Y.; Polgreen, P.M. Increased Statin Prescribing Does Not Lower Pneumonia Risk. *Clin. Infect. Dis.* **2015**, *60*, 1760–1766, https://doi.org/10.1093/cid/civ190.
26. Brooks, J.M.; Chrischilles, E.A.; Landrum, M.B.; Wright, K.B.; Fang, G.; Winer, E.P.; Keating, N.L. Survival Implications Associated with Variation in Mastectomy Rates for Early-Staged Breast Cancer. *Int. J. Surg. Oncol.* **2012**, *2012*, 1–9, https://doi.org/10.1155/2012/127854.
27. Brooks JM, Cook E, Chapman CG, et al. Statin Use after Acute Myocardial Infarction by Patient Complexity: Are the Rates Right? Med Care. 2015;53(4):324-331.
28. Brooks JM, Cook EA, Chapman CG, et al. Geographic Variation in Statin Use for Complex Acute Myocardial Infarction Patients: Evidence of Effective Care? Med Care. 2014;52 Suppl 3:S37-44.
29. Brooks, J.M.; Tang, Y.; Chapman, C.G.; Cook, E.A.; Chrischilles, E.A. What is the effect of area size when using local area practice style as an instrument?. *J. Clin. Epidemiology* **2013**, *66*, S69–S83, https://doi.org/10.1016/j.jclinepi.2013.04.008.
30. Brooks JM, Tang Y, Chapman CG, Cook EA, Chrischilles EA. What Is the Effect of Area Size When Using Local Area Practice Style as an Instrument? J Clin Epidemiol. 2013;66(8 Suppl):S69-83.
31. Fang G, Brooks JM, Chrischilles EA. A New Method to Measure Geographic Variation in Prescription Use and Its Implications for Comparative Effectiveness Research. Med Care. 2010;48:710-717.
32. Brooks JM, Chrischilles EA, Landrum MB, et al. Survival Implications Associated with Variation in Mastectomy Rates for Early-Staged Breast Cancer International Journal of Surgical Oncology. 2012;forthcoming.
33. Floyd, S.B.; Thigpen, C.; Kissenberth, M.; Brooks, J.M. Association of Surgical Treatment With Adverse Events and Mortality Among Medicare Beneficiaries With Proximal Humerus Fracture. *JAMA Netw. Open* **2020**, *3*, e1918663–e1918663, https://doi.org/10.1001/jamanetworkopen.2019.18663.
34. Fang, G.; Brooks, J.M.; Chrischilles, E. A New Method to Isolate Local-Area Practice Styles in Prescription Use as the Basis for Instrumental Variables in Comparative Effectiveness Research. *Med Care* **2010**, *48*, 710–717, https://doi.org/10.1097/mlr.0b013e3181e41bb2.
35. Fang, G.; Brooks, J.M.; Chrischilles, E.A. Comparison of Instrumental Variable Analysis Using a New Instrument With Risk Adjustment Methods to Reduce Confounding by Indication. *Am. J. Epidemiology* **2012**, *175*, 1142–1151, https://doi.org/10.1093/aje/kwr448.
36. Bucholz EM, Krumholz HA, Krumholz HM. Underweight, Markers of Cachexia, and Mortality in Acute Myocardial Infarction: A Prospective Cohort Study of Elderly Medicare Beneficiaries. PLoS Med. 2016;13(4):e1001998.
37. McAlister, F.A.; Oreopoulos, A.; Norris, C.; Graham, M.M.; Tsuyuki, R.T.; Knudtson, M.; Ghali, W.A. Exploring the Treatment-Risk Paradox in Coronary Disease. *Arch. Intern. Med.* **2007**, *167*, 1019–1025, https://doi.org/10.1001/archinte.167.10.1019.
38. Schneeweiss, S. Sensitivity analysis and external adjustment for unmeasured confounders in epidemiologic database studies of therapeutics. *Pharmacoepidemiol. Drug Saf.* **2006**, *15*, 291–303, https://doi.org/10.1002/pds.1200.
39. Özcan, C.; Deleskog, A.; Olsen, A.-M.S.; Christensen, H.N.; Hansen, M.L.; Gislason, G. Coronary artery disease severity and long-term cardiovascular risk in patients with myocardial infarction: a Danish nationwide register-based cohort study. *Eur. Hear. J. - Cardiovasc. Pharmacother.* **2017**, *4*, 25–35, https://doi.org/10.1093/ehjcvp/pvx009.
40. A Cook, E.; Schneider, K.M.; Robinson, J.; Wilwert, J.; Chrischilles, E.; Pendergast, J.; Brooks, J. Field methods in medical record abstraction: assessing the properties of comparative effectiveness estimates. *BMC Heal. Serv. Res.* **2014**, *14*, 391, https://doi.org/10.1186/1472-6963-14-391.
41. Katz, S.; Downs, T.D.; Cash, H.R.; Grotz, R.C. Progress in Development of the Index of ADL. *Gerontologist* **1970**, *10*, 20–30, doi:10.1093/geront/10.1_Part_1.20.
42. Piccirillo JF, Tierney RM, Costas I, Grove L, Spitznagel J, Edward L. Prognostic Importance of Comorbidity in a Hospital-Based Cancer Registry. JAMA. 2004;291(20):2441-2447.

43. Washington University School of Medicine. Adult Comorbidity Evaluation-27. Comorbidity Data Collection Web site. http://otooutcomes.wustl.edu/portals/otooutcomes/PDFs/2013Comorbidity-Data-Collection-Form.pdf. Published 2003. Accessed August 6, 2019.
